# Synthesis and Spectral Evaluation of Some Unsymmetrical Mesoporphyrinic Complexes

**DOI:** 10.3390/ijms13078112

**Published:** 2012-06-29

**Authors:** Rica Boscencu, Anabela Sousa Oliveira, Diana P. Ferreira, Luís Filipe Vieira Ferreira

**Affiliations:** 1 Faculty of Pharmacy, “Carol Davila” University of Medicine and Pharmacy, 6 Traian Vuia St., Bucharest 020956, Romania; 2 Molecular Physical Chemistry Center, IN—Institute of Nanoscience and Nanotechnology, Technical University of Lisbon, Av. Rovisco Pais, Lisbon 1049-001, Portugal; E-Mails: asoliveira@estgp.pt (A.S.O.); diana.ferreira@ist.utl.pt (D.P.F.); luisfilipevf@ist.utl.pt (L.F.V.F.); 3 School of Technology and Business, Polytechnic Institute of Portalegre, Lugar da Abadessa, Apartado 148, Portalegre 7301-901, Portugal

**Keywords:** unsymmetrical mesoporphyrinic complexes, microwave-assisted synthesis, solvatochromism, singlet oxygen, fluorescence quantum yields

## Abstract

Synthesis and spectral evaluation of new zinc and copper unsymmetrical mesoporphyrinic complexes are reported. Zn(II)-5-(4-acetoxy-3-methoxyphenyl)-10,15,20- tris*-*(4-carboxymethylphenyl)porphyrin, Zn(II)-5-[(3,4-methylenedioxy)phenyl]-10,15,20- tris*-*(4-carboxymethylphenyl)porphyrin, Cu(II)-5-(4-acetoxy-3-methoxyphenyl)-10,15,20- tris*-*(4-carboxymethylphenyl)porphyrin and Cu(II)-5-[(3,4-methylenedioxy)phenyl]-10,15,20- tris*-*(4-carboxymethylphenyl)porphyrin were synthesized using microwave-assisted synthesis. The complexes were characterized by elemental analysis, FT-IR, UV-Vis, EPR and NMR spectroscopy, which fully confirmed their structure. The spectral absorption properties of the porphyrinic complexes were studied in solvents with different polarities. Fluorescence emission and singlet oxygen formation quantum yields were evaluated for the compounds under study, revealing high yields for the zinc derivatives. The copper complexes are not emissive and only display residual capacity for singlet oxygen formation.

## 1. Introduction

Metalloporphyrins form an important class of tetrapyrrolic compounds which play a very important role in the metabolism of living organisms [1,2]. In addition, metalloporphyrins have been investigated for their application, one of the most promising being the diagnosis and treatment of malignant tumors [3–8]. Therefore, the synthesis and spectral evaluation of the porphyrinic structures are of current interest.

Photodynamic therapy involves selective accumulation of a photosensitizer in the tumor tissue, which, when activated with visible light in the presence of molecular oxygen results in reactive species production and consequently cellular destruction [9–11]. Photodynamic diagnosis is a detection procedure of molecular and/or physiological changes based on the accumulation of the photosensitizer at a specific target, which emits fluorescence upon light excitation [12–14]. In diagnostic and therapeutic applications the biomedical efficiency of the porphyrinic structures depends on their structural and physical–chemical characteristics.

The higher absorption coefficients in the spectral range 600–680 nm, high singlet oxygen generation quantum yield, acceptable solubility in biologic fluids for an easy localization at the cellular and subcellular level, great selectivity for the malignant or other targeted tissue, photostability and lack of toxicity in the absence of the exciting light are among the most important parameters that determine the efficiency of a photosensitizer [15–23].

The necessity to ensure a good localization of the porphyrinic complexes at the cellular level justifies the synthesis of unsymmetrical mesoporphyrinic complexes. This is possible by modifying the charge density and its distribution at the periphery of the tetrapyrrolic macrocycle. The compounds synthesized in this paper exhibit an unsymmetrical charge distribution over the macrocycle periphery of the tetrapyrrolic ring, therefore these molecules present a slight amphoteric character which favors the transport of the photosensitizer to the cellular targets, both in polar (the external environment of the cell) and nonpolar (the lipidic double layer of the cellular membrane) media.

The approach used in our previous studies for the synthesis and spectral characterization of several asymmetric mesoporphyrinic complexes was justified by the importance of this kind of compound for photodiagnosis and photodynamic therapy of malignant tumours.

In our previous papers we reported the synthesis of unsymmetrical mesoporphyrinic complexes by classical methods that involve refluxing of equimolar ratios of the porphyrinic ligands and metal salts in the presence of a basic catalyst [24,25]. This requires as a first step preparation of the porphyrinic ligand by condensation of pyrrole and substituted benzaldehydes in acidic medium and at ~140 °C. Under these conditions, chlorins (reduced forms of the porphyrins) result as secondary products of the reaction and isolation of mesoporphyrinic compound requires tedious chromatographic separations with decreasing reaction yield.

In this paper, new unsymmetrical mesoporphyrinic complexes with a high degree of purity were obtained by a novel ecological, efficient and versatile synthetic method as an alternative to the classical way.

**Figure 1:**
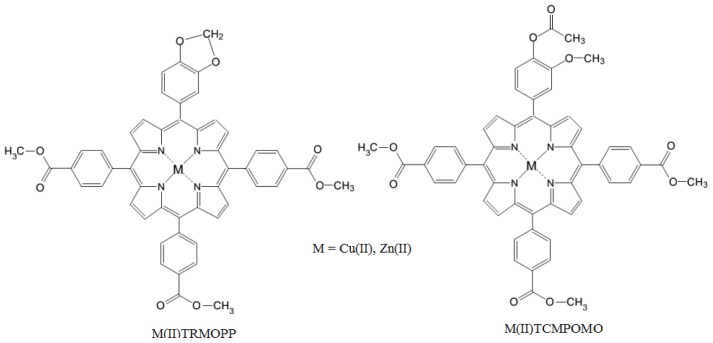
General structures of the unsymmetrical mesoporphyrinic complexes.

Zn(II)-5-(4-acetoxy-3-methoxyphenyl)-10,15,20-tris-(4-carboxymethylphenyl)porphyrin (Zn(II)TCMP OMO), Zn(II)-5-[(3,4-methylenedioxy)phenyl]-10,15,20-tris-(4-carboxymethylphenyl)porphyrin (Zn(II)TRMOPP), Cu(II)-5-(4-acetoxy-3-methoxyphenyl)-10,15,20-tris-(4-carboxymethylphenyl) porphyrin (Cu(II)TCMPOMO) and Cu(II)-5-[(3,4-methylenedioxy)phenyl]-10,15,20-tris-(4-carboxy methylphenyl)porphyrin (Cu(II)TRMOPP) (Figure 1) were synthesized using microwave-assisted synthesis in order to reduce the reaction time, increase reaction yield and provide the needed degree of purity of the mesoporphyrinic complexes. In addition, the specific conditions for initiating synthesis by microwave irradiation (absence of acidic medium, moderate temperatures) eliminate the formation of chlorins and allow an easier purification of the mesoporphyrinic complexes.

Considering the fact that tetrapyrrolic complexes exhibit bioactivity only after the cell membrane permeation, the study of their spectral properties in environments with different polarities is very important for their biomedical applications. In this paper, the solvatochromic behavior of the Soret and Q bands of the complexes was carried out using a series of organic solvents: ethanol, isopropyl alcohol, chloroform, dimethyl sulfoxide and dimethylformamide. In addition, a study of the fluorescence emission and singlet oxygen formation quantum yields for unsymmetrical mesoporphyrinic complexes was performed.

## 2. Results and Discussion

Microwave-assisted synthesis of the porphyrinic compounds is increasingly applied as an alternative method to the classic way, due to certain advantages, particularly the shorter reaction times, higher reaction yields, absence of solvent from the reaction mixture and increased selectivity of the synthesis reactions [26–34].

In this paper, the synthesis of the mesoporphyrinic complexes was carried out using microwave irradiation of the reaction mixture consisting in substituted benzaldehyde, pyrrole and metallic salt in the presence of the basic catalyst in dry media. The synthesis reactions have been successfully repeated several times with identical results and then the porphyrinic complexes were characterized by elemental analysis, IR, UV-Vis, NMR and EPR spectrometry. Special attention was devoted to the study of the fluorescence emission and singlet oxygen formation quantum yields for the compounds under study.

### 2.1. Infrared Spectra

The main infrared absorption bands of the mesoporphyrinic complexes presented in this work with their assignments are listed in Table 1. These assignments are generally in agreement with those previously reported for similar structures [35,36]. The medium intensity band at about 2850 cm^−1^ can be assigned to C–H vibration frequencies of the –O–CH_3_ group. The band observed at ~2922 cm^−1^ was assigned to C–H vibration of the phenyl groups. Also, for Zn(II)TRMOPP and Cu(II)TRMOPP the signal at ~2915 cm^−1^ can be attributed to the stretching vibration of C–H from –O–CH_2_–O– group.

The IR bands located in the spectral range of 1490–1510 cm^−1^ are assigned to C=N stretching vibration and the bands in the range of 1602–1606 cm^−1^ are assigned to C–N vibrations of the porphyrinic core. Also, in the infrared spectra of the copper and zinc porphyrinic complexes a band corresponding to C=O vibration frequencies at ~1723 cm^−1^ and another at ~1190 cm^−1^ corresponding to C–O bond vibrations was highlighted. Other bands observed in the infrared spectrum of the complexes are due to the vibrational motion of C–H bond of the pyrrole ring and were identified at ~1460 cm^−1^. Also, the medium intensity bands at about 1000 cm^−1^ were attributed to C–H deformation vibrations.

### 2.2. Absorption Spectra

The complexes were studied by UV-Vis spectroscopy to confirm their structure and behavior in environments with different polarities.

**Figure 2:**
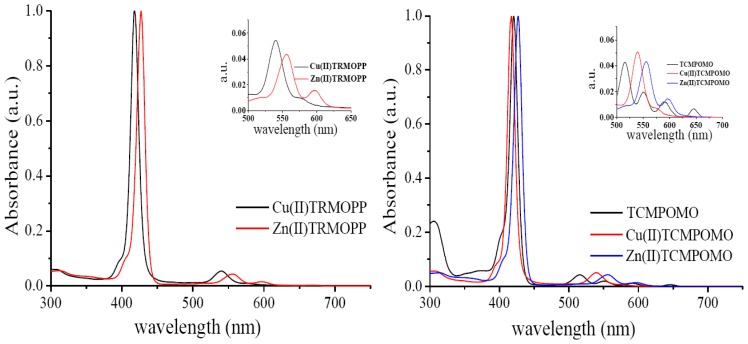
Normalized UV-Vis spectra of Cu(II)TRMOPP, Zn(II)TRMOPP, TCMPOMO, Cu(II)TCMPOMO and Zn(II)TCMPOMO porphyrins in chloroform.

The analysis of the porphyrinic complexes by UV-Vis spectroscopy is an efficient method for their identification because their molecular absorption spectra contain a Soret (B) band situated in the spectral range 400–440 nm, one or two Q bands located between 500 and 650 nm for metallated porphyrins and four Q bands for metal free porphyrins as can be seen in Figure 2. According to Gouterman’s theory, the Soret band is the result of a_1u_ (π)→e_g_ (π*) transition and Q bands corresponding to the a_2u_ (π)→e_g_ (π*) transition [37,38]. The UV-Vis spectral data (Table 2) reveals for the synthesized complexes one Soret band (413–432 nm) and one or two Q bands (537–603 nm) characteristic of metalloporphyrins.

The main differences observed in the absorption characteristics of the complexes presented in this work are determined by the nature of the metallic ion and environmental polarity. Thus, depending on solvent polarity, UV-Vis spectrum of the Zn(II) complexes displayed one Soret band, between 419 and 432 nm and two Q bands in the 546–603 nm spectral range. Also, for the copper complexes the UV-Vis spectrum revealed a Soret band in the wavelength region 413–421 nm and one Q band between 537 and 545 nm.

Comparing the UV-Vis spectra corresponding to the zinc porphyrinic complexes *versus* copper porphyrinic complexes (as can be seen in Table 2) the blue shift of the spectral bands of the copper complex was evident. In agreement with Gouterman’s theory, this blue shift of the Soret and Q bands could originate from the stronger conjugation effects that occur between the Cu(II) orbitals and the π electrons of the tetrapyrrolic ring, effects that cause a decrease of the energy of the a_1u_ (π) and a_2u_ (π) orbitals relative to the e_g_ (π*) orbitals with increased energy available for electron transitions [38]. The analysis of the spectral data obtained for the mesoporphyrinic complexes in solvents with different polarities (Table 2) found that a decrease of solvent polarity causes batochromic shifts of the spectral bands. These spectral changes can be ascribed to the formation of proton bridges between the solvent molecules and the porphyrinic substitutents. The spectral properties of the unsymmetrically synthesized complexes are quite similar to the corresponding symmetrical compounds, both in shape and in the ratio between the molar absorptivities of the bands [35,36]. Therefore, changing the degree of symmetry produces no significant change of UV-Vis absorbtion properties.

### 2.3. Fluorescence Emissions, Lifetimes and Singlet Oxygen Formation

All the significant parameters, including singlet oxygen formation quantum yields for the mesoporphyrinic compounds under study are summarized in Table 3.

**Figure 3:**
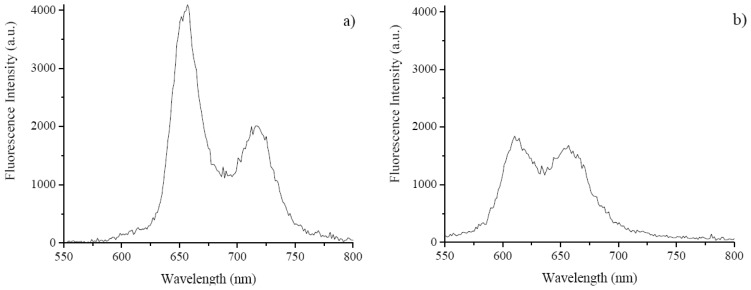
Fluorescence spectra of TCMPOMO (**a**) and Zn(II)TCMPOMO (**b**) in chloroform.

**Figure 4:**
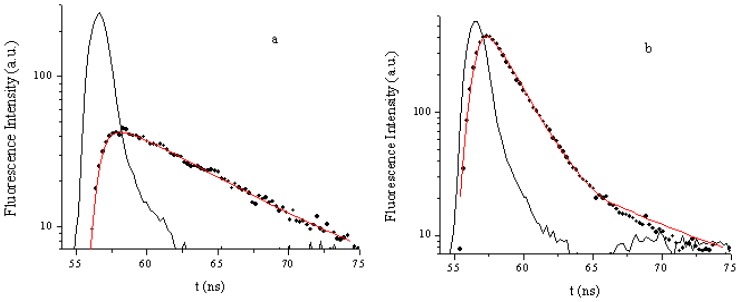
Fluorescence lifetimes decays of TCMPOMO (**a**) and Zn(II)TCMPOMO (**b**) in chloroform (black line—ludox, black points—sample and red line—fitting).

**Figure 5:**
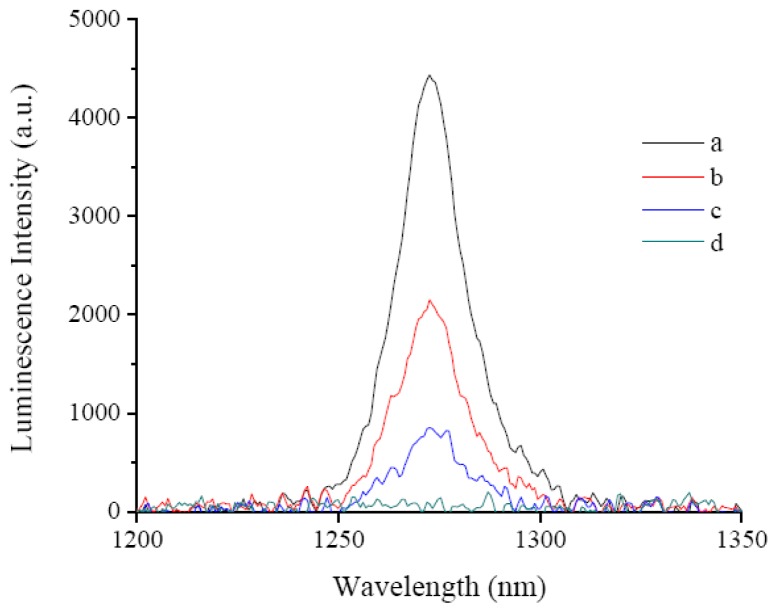
Singlet Oxygen Emission Spectra of Phenazine (**a**), TCMPOMO (**b**), Zn(II)TCMPOMO (**c**) and Cu(II)TCMPOMO (**d**) in chloroform.

The laser induced fluorescence emission of TCMPOMO and Zn(II)TCMPOMO (Cu(II)TCMPOMO is not emissive) in chloroform is presented in Figure 3. Figure 4 presents the lifetime decays for the same compounds and Figure 5 illustrates the singlet oxygen emission spectra of the same compounds in chloroform, with Phenazine as a reference spectrum.

The incorporation of Zn(II) in the porphyrinic ring influenced the fluorescence emission of the Zn(II)TCMPOMO in both its general shape and the position of the bands. Namely, the higher band of TCMPOMO is reduced to approximately one half in the presence of the metal ion, while the second band remains at approximately the same intensity. Also, a hypsochromic shift is present in the case of the metallated compound compared to the free-base.

The data of Table 3 shows that Cu(II) inclusion in the porphyrinic ring eliminates the fluorescence emission. This effect is due to the copper presence which increases the non-radiative decay of the excited singlet state of the porphyrin [40]. At the same time the quantum yield of singlet oxygen formation, which involves the triplet excited state of the porphyrin also decreases. Both metal free and zinc porphyrins have good singlet oxygen quantum yields of formation that may enable efficient attack of sensitive cell components such as membranes and nucleic acids, pointing to the use of these compounds as phototoxic agents that might be important as cancer photodynamic therapy drugs.

Metal free porphyrin exhibits a fluorescence quantum yield of emission of Φ_F_ = 0.09 (the reference for the fluorescence quantum yield determination was TPP in CHCl_3_ (Φ_F_ = 0.11)). Similar results were obtained by the use of Zn(II)TPP as a secondary reference (Φ_F_ = 0.04). TCMPOMO also exhibits a long lifetime (τ_F_ = 7.9 ns), which indicates that this compound can be used as a diagnostic tool for the detection of cancer cells [20].

## 3. Experimental Section

### 3.1. Materials and Methods

Commercially available chemicals and solvents were used as received from Sigma-Aldrich and Merck. For the microwave assisted synthesis we used a CLATRONIC MWG775 H type temperature-controlled microwave oven.

The elemental analysis of C, H and N was performed with an automatic Carlo Erba 1108 analyzer. IR spectra were recorded with a FT-IR 400D Nicolet Impact spectrophotometer. The substances under analysis, previously dried for 24 h at 150 °C, were processed as KBr pellets. The spectra were recorded in the 4000–500 cm^−1^ spectral range.

The NMR spectra of the zinc porphyrinic complex were recorded with a 400 MHz Bruker NMR Spectrometer. EPR spectra of the copper porphyrinic complex were recorded on powders at room temperature using an ART-6 spectrometer, operating in the X band (9.01 GHz), equipped with a field modulation unit of 100 KHz.

The absorption spectra of the mesoporphyrinic complexes were recorded with the use of a Lambda 35 Perkin-Elmer spectrophotometer in different media (ethanol, isopropyl alcohol, dimethylformamide, chloroform, dimethyl sulfoxide) using a 10 mm path length quartz cell, in single beam mode.

The metalloporphyrin solutions were freshly prepared in the spectrally pure solvents at the concentration 2.5 × 10^−6^ M and kept in dark until the measurement to prevent photodegradation.

Fluorescence lifetimes were determined using Easylife VTM equipment from OBB (Lifetime range from 100 ps to 3 μs). This technique uses pulsed light sources from different LEDs (310 nm in this case) and measures fluorescence intensity at different time delays after the excitation pulse. In this case, 590 nm cut-off filters were used at emission both for solution and for solid samples, depending on the sample under study. The instrument response function was measured using a Ludox scattering solution. FelixGX software from OBB was used for fitting and analysis of the decay dynamics, 1 to 4 exponentials and also a lifetime distribution analysis [41], the Exponential Series Method (ESM).

The schematic diagram of the LIL system is presented in reference [42]. Briefly, a N_2_ laser (PTI model 2000, *ca.* 600 ps FWHM, ~1.0 mJ per pulse), was used in laser-induced luminescence experiments. In this case the excitation wavelength was 337 nm. The light arising from the irradiation of the samples by the laser pulse was collected by a collimating beam probe coupled to an optical fiber (fused silica) and detected by a gated intensified charge coupled device Andor ICCD, model i-Star 720. The ICCD was coupled to a fixed compact imaging spectrograph (Andor, model Shamrock 163). The system can be used either by capturing all light emitted by the sample or in a time-resolved mode. The ICCD has high speed gating electronics (about 2.3 ns) and intensifier and cover at least the 250–950 nm wavelength range. Time-resolved absorption and emission spectra are available in a time range from nanoseconds to seconds. With this set-up, both fluorescence and phosphorescence spectra were easily available by the use of the variable time gate width and start delay facilities of the ICCD.

The singlet oxygen measurement set-up was assembled in our laboratory. As an excitation source we use the nitrogen laser. The detector is an InGaAs CCD (model i-Dus from Andor) working at low temperature (−60 °C) coupled to a fixed spectrograph, model Shamrock 163i also from Andor. Long pass filters were used to exclude totally avoid the excitation radiation from reaching the detector (LFP1000 or LFP1100 from CVI Lasers). By comparing the total area of the emission spectra for the reference and also for the samples under study in the same solvent, with the same optical density at the excitation wavelength, the φ_Δ_ values were obtained.

### 3.2. Synthesis of Zn(II)-5-(4-Acetoxy-3-methoxyphenyl)-10,15,20-tris-(4-carboxymethylphenyl)porphyrin (Zn(II)TCMPOMO) and Zn(II)-5-[(3,4-Methylenedioxy)phenyl]-10,15,20-tris-(4-carboxymethylphenyl) porphyrin (Zn(II)TRMOPP)

A mixture of 4-acetoxy-3-methoxybenzaldehyde (1.94 g, 0.01 mol), methyl 4-formyl benzoate (4.92 g, 0.03 mol), pyrrole (2.76 mL, 0.04 mol), anhydrous zinc acetate (1.83 g, 0.01 mol) and 2–3 g of silica gel 60 (200–500 μm, 35–70 mesh) in the presence of 2,6-dimethylpyridine (1 mL) was subjected to microwave irradiation at 400 W for 10 min. Extraction of samples for monitoring the synthesis by thin layer chromatography and UV-Vis spectroscopy was performed after every 2 min of irradiation. Thin layer chromatography (dichloromethane/diethyl ether 50:1 v/v) of the crude product of reaction revealed presence of a mixture six metalloporphyrin isomers (A_4_, A_3_B, A_2_B_2_ (*cis* and *trans*), AB_3_ and B_4_-type) with high content of A_3_B isomer (Zn(II)-5-(4-acetoxy-3-methoxyphenyl)-10,15,20-tris-(4- carboxymethylphenyl)porphyrin).

The reaction product was extracted with dichloromethane/diethyl ether (50:1, v/v). The extract was filtered, the solvent was removed under vacuum and the product was purified by column chromatography, using silica gel (100–200 mesh size) as stationary phase and dichloromethane/diethyl ether (50:1, v/v) as eluent.

The compound of interest presents a violet color and was the second band that passes through the chromatographic column. The solution of the zinc complex was concentrated by simple distillation.

The product obtained as violet crystals was dried at ≈100 °C for 12 h. Zn (II)-5-(4-acetoxy-3- methoxyphenyl)-10,15,20-tris*-*(4-carboxymethylphenyl)porphyrin was obtained with a yield of 43%. Elemental analysis for C_53_H_38_N_4_O_9_Zn: calculated C 67.73, H 4.04, N 5.96; found C 67.62, H 3.98, N 5.83. The chemical shifts of the NMR signals for the Zn(II)TCMPOMO are as follows: ^1^H-NMR, *δ*_H_ (400 MHz, CDCl_3_), ppm: 3.61 (s, 3H), 3.92 (s, 3H), 4.13 (s, 9H), 7.09 (d, *J* = 8.5 Hz, 1H), 7.25 (s, 1H), 7.45 (d, *J* = 8.5 Hz, 1H), 8.34 (d, *J* = 8.0 Hz, 6H), 8.48 (d, *J* = 8.0 Hz, 6H), 8.87 (d, *J* = 4.70 Hz, 6H), 8.98 (d, *J* = 4.72 Hz, 2H). ^13^C-NMR *δ*_C_ (400 MHz, CDCl_3_), ppm: 52.3, 74.0, 76.7, 106.7, 115.8, 119.8, 120.1, 127.9, 128.0, 128.8, 129.3, 130.0, 134.5, 135.8, 144.0, 146.3, 147.7.

The same procedure was adopted in the preparation of Zn(II)TRMOPP (yield 40%) and the following results were obtaining. Elemental analysis for C_51_H_34_N_4_O_8_Zn: calculated C 68.38, H 3.79, N 6.25; found C 68.21, H 3.65, N 6.08. The chemical shifts of the NMR signals for the Zn(II)TRMOPP are as follows: ^1^H-NMR, *δ*_H_ (400 MHz, CDCl_3_), ppm: 4.13 (s, 9H), 6.30 (s, 2H), 7.28 (d, 1H), 7.66 (d, 1H), 7.72 (s, 1H), 8.32 (d, *J* = 8.0 Hz, 6H), 8.48 (d, *J* = 8.0 Hz, 6H), 8.89 (d, *J* = 4.70, 6H), 8.92 (d, *J* = 4.70Hz, 2H). ^13^C-NMR, *δ*_C_ (400 MHz, CDCl_3_), ppm: 52.4, 76.7, 101.5, 106.9, 115.1, 119.1, 120.1, 127.9, 128.0, 128.6, 129.7, 130.1, 134.4, 135.4, 143.5, 146.3, 147.7.

### 3.3. Synthesis of Cu(II)-5-(4-Acetoxy-3-methoxyphenyl)-10,15,20-tris-(4-carboxymethylphenyl)porphyrin (Cu(II)TCMPOMO) and Cu(II)-5-[(3,4-Methylenedioxy)phenyl]-10,15,20-tris-(4-carboxymethylphenyl) porphyrin (Cu(II)TRMOPP)

A mixture of anhydrous copper(II) chloride (1.34 g, 0.01 mol), methyl 4-formyl benzoate (4.92 g, 0.03 mol), 4-acetoxy-3-methoxybenzaldehyde (1,94 g, 0.01 mol), pyrrole (2.76 mL, 0.04 mol), 2–3 g of silica gel 60 (200–500 μm, 35–70 mesh) and 2,6-dimethylpyridine (1 mL) was irradiated in a microwave oven at 475 W for 8 min. The extent of the complexation reaction was monitored by thin layer chromatography and UV-Vis spectroscopy. For this purpose, extraction of samples was performed after every 2 min of irradiation. TLC test of the final product of microwave-assisted reaction revealed presence of a six metalloporphyrin isomers (A_4_, A_3_B, A_2_B_2_ (*cis* and *trans*), AB_3_ and B_4_-type) with high content of A_3_B isomer.

The crude product was dissolved in dichloromethane/diethyl ether (50:1, v/v), filtered and finally purified on a chromatography column by repeated elution, using silica gel (100–200 mesh size) as stationary phase and dichloromethane/diethyl ether (50:1, v/v) as eluent.

The compound of interest was the second band passing through the chromatographic column. The solution of the asymmetrical copper porphyrinic complex was concentrated by simple distillation. The obtained dark red crystals were dried at ≈100 °C for 12 h. Cu(II)-5-(4-acetoxy-3-methoxyphenyl)- 10,15,20-tris-(4-carboxymethylphenyl)porphyrin was obtained with a yield of 47%. Elemental analysis for C_53_H_38_N_4_O_9_Cu: calculated C 67.84, H 4.05, N 5.97; found C 67.70, H 3.95, N 5.86.

The preparation of the Cu(II)TRMOPP was similar to that of Cu(II)TCMPOMO with a yield of 42%. Elemental analysis for C_51_H_34_N_4_O_8_Cu: calculated C 68.49, H 3.80, N 6.26; found C 68.36, H 3.71, N 6.12.

EPR spectra of the Cu(II)TRMOPP and Cu(II)TCMPOMO recorded in solid state at room temperature provide information about the coordination environment around copper ion.

The EPR parameters evaluated for the copper porphyrinic complexes are: Cu(II)TCMPOMO g_||_ = 2.200, g_⊥_ = 2.055, α^2^ = 0.6926, A_||_ = 156.953 × 10^−4^cm^−1^ and g_||_ = 2.175, g_⊥_ = 2.05, α^2^ = 0.6946, A_||_ = 202 × 10^−4^cm^−1^ for Cu(II)TRMOPP. These values are close to those reported in the literature for copper porphyrins and confirm a square planar geometrical arrangement of nitrogen atoms around the copper ion [43,44].

According to Kivelson and Neiman the α^2^ and g_||_ values indicates a covalent character of the Cu–N bonds in the copper porphyrinic complex [45].

## 4. Conclusions

The paper describes the synthesis and spectral studies of the Zn(II)-5-(4-acetoxy-3- methoxyphenyl)-10,15,20-tris-(4-carboxymethylphenyl)porphyrin, Zn(II)-5-[(3,4-methylenedioxy) phenyl]-10,15,20-tris-(4-carboxymethylphenyl)porphyrin, Cu(II)-5-(4-acetoxy-3-methoxyphenyl)-10, 15,20-tris-(4-carboxymethylphenyl)porphyrin and Cu(II)-5-[(3,4-methylenedioxy)phenyl]-10,15, 20-tris-(4-carboxymethylphenyl)porphyrin.

The complexes were obtained in a short time, with good yields using an ecological method of synthesis. The structures of metalloporphyrins were confirmed by elemental analysis, UV-Vis, FT-IR, NMR and EPR spectroscopy.

The influence of environment polarity on spectral absorption properties was investigated. UV-Vis spectra of the complexes in different solvents resulted in red shift of Soret and Q bands with increasing environmental polarity due to the proton bridges between the solvent molecules and the porphyrinic substituents.

At the concentrations used in this study (ranging from *c* = 5.0 × 10^−7^ M to *c* = 5.0 × 10^−6^ M) and for all samples used, no aggregation effects were found, judging from the complete superposition of the absorption spectra obtained in the above mentioned concentration range. Fluorescence emission spectra also provided evidence of reduced re-absorption effects, in the range of concentrations mentioned before.

Fluorescence emission quantum yields evaluated for porphyrinic complexes revealed high yields for the zinc derivative. The copper complexes are not emissive. A similar pattern was detected for singlet oxygen quantum yields of formation. These latter values are a good indication for their use as possible photosensitizers for photodynamic therapy.

## Figures and Tables

**Table 1:** Infrared spectral assignments of the unsymmetrical mesoporphyrinic complexes (cm^−1^).

Assignments	Zn(II)TCMPOMO	Cu(II)TCMPOMO	Zn(II)TRMOPP	Cu(II)TRMOPP
**ν_C–H_**	2920*m*	2921*m*	2922*w*	2924*w*
**ν_C–H from –O–CH2–O–_**	-	-	2915*m*	2916*m*
**ν_C–H from –O–CH3_**	2850*m*	2851*m*	2850*m*	2850*m*
**ν_C = O_**	1723*m*	1724*m*	1722*m*	1725*m*
**ν_C–N_**	1606*m*	1602*m*	1605*m*	1606*m*
**ν_C = N_**	1510*m*	1500*m*	1492*m*	1496*m*
**ν_C–H pyrrole_**	1456*w*	1458*w*	1433*w*	1433*w*
**ν_C–O_**	1190*s*	1188*m*	1198*s*	1195*s*
**δ_C–H_**	997*m*	1000*m*	996*m*	999*m*
**γ_C–C_**	867*w*	865*w*	867*w*	867*w*
**γ_C–N pyrrole_**	795*m*	799*m*	780*m*	792*m*

The intensities of the signals are described as weak (*w*), medium (*m*), strong (*s*).

**Table 2:** Wavelengths maxima (λ_max_) and molar absorptivity values (lg ɛ) for the mesoporphyrinic complexes in different solvents (*c* = 2.5 × 10^−6^ M).

Solvent	λ_max_ (nm) [lg ɛ (L·mol^−1^·cm^−1^)]		

	Soret band B(0,0)	Q bands Q_y_(0,0)	Q_x_(1,0)
Zn(II)-5-(4-acetoxy-3-methoxyphenyl)-10,15,20-tris-(4-carboxymethylphenyl)porphyrin			
EtOH	426.2 [5.646]	558.6 [4.203]	599.3 [3.783]
iso-PrOH	427.0 [5.641]	558.6 [4.271]	599.1 [3.932]
CHCl_3_	422.6 [5.584]	549.8 [4.261]	589.5 [3.690]
DMF	428.9 [5.602]	560.3 [4.201]	601.4 [3.914]
DMSO	431.5 [5.588]	562.0 [4.227]	602.7 [3.979]
Cu(II)-5-(4-acetoxy-3-methoxyphenyl)-10,15,20-tris-(4-carboxymethylphenyl)porphyrin			
EtOH	413.0 [5.629]	537.0 [4.095]	-
iso-PrOH	414.1 [5.507]	537.1 [4.174]	-
CHCl_3_	416.3 [5.539]	539.9 [4.325]	-
DMF	417.0 [5.565]	540.1 [4.142]	-
DMSO	420.8 [5.479]	544.0 [4.261]	-
Zn(II)-5-[(3,4-methylenedioxy)phenyl]-10,15,20-tris-(4-carboxymethylphenyl)porphyrin			
EtOH	426.0 [5.687]	558.2 [4.292]	598.7 [3.924]
iso-PrOH	426.3 [5.671]	558.4 [4.274]	598.7 [3.903]
CHCl_3_	422.0 [5.572]	549.3 [4.271]	587.2 [3.680]
DMF	429.0 [5.635]	559.8 [4.255]	601.0 [3.944]
DMSO 1	431.0 [5.632]	561.9 [4.246]	603.0 [3.964]
Cu(II)-5-[(3,4-methylenedioxy)phenyl]-10,15,20-tris-(4-carboxymethylphenyl)porphyrin			
EtOH	414.1 [5.778]	538.0 [4.447]	-
iso-PrOH	414.1 [5.772]	538.0 [4.435]	-
CHCl_3_	416.2 [5.698]	539.4 [4.387]	-
DMF	418.2 [5.668]	540.8 [4.394]	-
DMSO 1	422.3 [5.652]	544.4 [4.408]	-

EtOH = ethanol, iso-PrOH = isopropyl alcohol, CHCl_3_ = chloroform, DMF = dimethylformamide, DMSO = dimethyl sulfoxide.

1 Data were taken from [39].

**Table 3:** Fluorescence emission quantum yields, singlet oxygen formation quantum yields and fluorescence lifetimes for the mesoporphyrinic compounds in chloroform.

Porphyrinic compounds	Φ_Δ_	Φ_F_	τ_F_ (ns)
TCMPOMO	0.42	0.09	7.89
Cu(II)TCMPOMO	0.08	<0.01	**1.90**
Zn(II)TCMPOMO	0.16	0.06	1.75
Cu(II)TRMOPP	0.04	<lod *	<lod *
Zn(II)TRMOPP	0.24	0.06	1.71

* minimum level of detection in our set-up.
